# Developing the PTSD Checklist-I/F for the DSM-IV (PCL-I/F): Assessing PTSD Symptom Frequency and Intensity in a Pilot Study of Male Veterans with Combat-Related PTSD

**DOI:** 10.3390/bs5010059

**Published:** 2015-02-03

**Authors:** Ryan Holliday, Julia Smith, Carol North, Alina Surís

**Affiliations:** 1 Veteran Affairs North Texas Health Care System, 4500 S. Lancaster Rd., Dallas, TX 75216, USA; E-Mails: ryan.holliday@utsouthwestern.edu (R.H.); julia.smith2@va.gov (J.S.); carol.north@utsouthwestern.edu (C.N.); 2 University of Texas Southwestern Medical Center, 5323 Harry Hines Blvd., Dallas, TX 75390, USA

**Keywords:** posttraumatic stress disorder, PTSD, screening, assessment, PTSD Checklist, PCL, PCL-I/F, combat-related PTSD, male veterans, PTSD symptom frequency

## Abstract

The widely used posttraumatic stress disorder (PTSD) Checklist (PCL) has established reliability and validity, but it does not differentiate posttraumatic symptom frequency from intensity as elements of posttraumatic symptom severity. Thus, the PCL in its existing form may not provide a comprehensive appraisal of posttraumatic symptomatology. Because of this, we modified the PCL to create the PCL-I/F that measures both frequency and intensity of PTSD symptoms via brief self-report. To establish validity and internal consistency of the PCL-I/F, we conducted a pilot study comparing PCL-I/F scores to structured diagnostic interview for PTSD (the Clinician Administered PTSD Scale [CAPS]) in a male combat veteran sample of 92 participants. Statistically significant correlations between the PCL-I/F and the CAPS were found, suggesting initial validation of the PCL-I/F to screen and assess frequency and intensity of combat-related PTSD symptoms. Implications are discussed for screening and assessment of PTSD related to combat and non-combat trauma.

## 1. Introduction

Approximately 41% of the general population will experience at least one traumatic event in their lifetime [1]. Of this portion, approximately 5.6% of the general population will go on to develop posttraumatic stress disorder (PTSD) [2]. However, certain subsets of the general population have a greater incidence of traumatic events and PTSD. Veterans are one such group, who may experience more frequent and severe trauma (e.g., combat and wartime stressors) than the rest of the population, with a higher prevalence of PTSD [3]. For example, a review of the literature by Richardson *et al.* [3] estimated that up to 17% of combat veterans will develop PTSD in their lifetime.

To target and treat this specific population, the Veterans Health Administration has placed an emphasis on developing and implementing valid and reliable measures of PTSD and posttraumatic symptom severity [4]. A widely utilized posttraumatic symptom assessment tool is the PTSD Checklist (PCL) [5], which is a brief self-report questionnaire that measures posttraumatic symptom severity level. The popularity of the PCL lies in its well-documented validity, its measurement of the list of symptoms of PTSD in the *Diagnostic and Statistical Manual* of the American Psychiatric Association, and its ease of administration as a self-report instrument in a fraction of the time required by a full diagnostic interview such as the Clinician Administered PTSD Scale (CAPS) [6,7,8]. Versions of the PCL have been validated for use with military personnel or veterans and civilians, and for assessment of specific traumatic experiences [6,7,9].

The CAPS differentiates intensity from frequency characteristics of posttraumatic symptoms, rating them separately as two aspects of symptom severity [10]. The PCL, however, does not rate these two related constructs separately, instead assessing severity through the singular phrase “how much you have been bothered” by the symptoms on a 5-point scale rated from “not at all” to “extremely”. Both intensity and frequency of symptoms are aspects of severity. Therefore, a desired feature of the PCL would be the rating of intensity and frequency separately as components of symptom severity, in a brief self-report measure. These features are not currently available in any instrument. The success of the CAPS in measuring these two aspects of symptom severity suggests that it should be possible to successfully modify the PCL to provide data for both intensity and frequency of symptoms. Therefore, this study sought to develop and provide initial validation of a modified version of the PCL that measures both intensity and frequency of posttraumatic symptoms.

## 2. Method

### 2.1. Sample

A volunteer sample of 92 veterans with PTSD was recruited for a randomized clinical trial of dexamethasone for PTSD, through clinician referral, advertisements, and identification from a local voluntary research candidate database, at a large Southwestern Veteran Affairs Medical Center (VAMC) [11]. Potential participants were excluded if they had active psychosis, current major depressive disorder with melancholia, substance dependence in the last three months, acute suicidality or homicidality, or medical contraindications to administration of dexamethasone.

### 2.2. Procedures

The study was approved by the local VAMC Institutional Review Board and all participants provided written consent before taking part in the study. Monetary compensation was provided as an incentive for participation. The assessments presented in this article were administered at baseline after recruitment into the study.

### 2.3. Measures

#### 2.3.1. Development of the PCL-I/F

The PCL-I/F was created through modification of the PCL. The PCL is a 17-item self-report questionnaire that measures the severity of *Diagnostic and Statistical Manual of Mental Health Disorders, Fourth Edition* (*DSM-IV-TR*) PTSD symptoms in the past month [12] related to their “most distressing” specific military-related trauma experience. Each item is rated on a 5-point Likert scale ranging from 1 (“Not at all”) to 5 (“Extremely”). Symptom scores are provided for the total of symptoms as well as separately for symptom groups B (intrusive re-experience), C (avoidance and numbing), and D (hyperarousal). Symptoms rated 3 (“Moderately”) or higher are counted as indicative of clinically distressing symptoms. The PCL generates a total score of posttraumatic symptom severity, as well as symptom severity scores pertaining to the three PTSD DSM-IV symptom group criteria [12]: (1) criterion B (re-experiencing symptoms); (2) criterion C (avoidance and numbing symptoms); and (3) criterion D (hyperarousal symptoms). Internal consistency has been demonstrated to be strong for the PCL total symptom score (Cronbach’s α = 0.97) and for symptom group B, C, and D scores (Cronbach’s α = 0.92–0.93). The PCL also has very strong test-retest reliability (*r* = 0.96) and has strong concurrent validity to numerous measures for PTSD including the CAPS (*r* = 0.93) and Mississippi Scale for Combat PTSD (*r* = 0.70, *r* = 0.81) [6,13].

The PCL’s symptom rating procedure was expanded in constructing the PCL-I/F to enable separate rating of each symptom for frequency and for intensity. Separate PCL-Intensity (PCL-I) and PCL-Frequency (PCL-F) forms were created to allow construction of separate subscales for symptom frequency and intensity (see forms provided in Appendix). The PCL’s symptom rating instructions and the rating category titles were reworded to provide specific language eliciting separate frequency and intensity symptom ratings. Symptom intensity for the PCL-I scale was assessed “to what degree” the person was bothered by the symptom in the last month, with choices of “not at all”, “a little bit”, “moderately”, “quite a bit”, or “extremely” rated from 1 (for “not at all”) to 5 (for “extremely”). Symptom frequency for the PCL-F subscale was assessed by “how often” the person was bothered by the symptom in the last month, with choices of “not at all”, “once or twice”, “1–2 days/week”, “3–4 days/week”, and “daily or almost every day” rated from 1 (for “not at all”) to 5 (for “daily or almost every day”).

#### 2.3.2. Assessment Instruments

The newly-developed PCL-I/F and the CAPS were administered to all study participants at baseline. Participants were first administered the CAPS to confirm their PTSD diagnosis. Following this, each participant was then administered the PCL-I followed by the PCL-F.

PCL-I/F. This 34-item self-report questionnaire measures the severity of *DSM-IV-TR* PTSD symptoms in the past month related to the individual’s “most distressing” specific military-related trauma experience. Intensity and frequency of each symptom are rated on a 5-point Likert scale. The PCL-I/F yields separate subscores for intensity (range = 17–85) and frequency (range = 17–85) as well as an overall score (range = 34–170) representing the sum of these two subscales. Separate intensity and frequency subscores and total scores for symptom groups B, C, and D can also be produced. The measure requires only 5–10 min to administer and score. The psychometric properties of the PCL-I/F were tested as part of this study, and this information is presented in the Results section of this article.

CAPS. This 30-item semi-structured interview assesses both the intensity and frequency of the 17 *DSM-IV-TR* PTSD symptoms within the past month [8,10]. During the interview, a clinician rates the intensity and frequency of each symptom on a 5-point Likert scale ranging from 0 (intensity = “none”/frequency = “never”) to 4 (intensity = “extreme”/frequency = “daily or almost every day”). The CAPS also yields separate subscores for intensity (range = 0–68) and frequency (range = 0–68) as well as an overall score representing the sum of these two subscales (range = 0–132). Separate intensity and frequency subscores and total scores for symptom groups B, C, and D can also be produced. The CAPS has strong inter-rater reliability for total score as well as scores for intensity and frequency (*κ* = 0.95–1.00). The CAPS also has strong concurrent validity in studies comparing it to other commonly used measures of PTSD including the PCL (*r* = 0.93) and Mississippi Scale for Combat-related PTSD (*r* = 0.70, *r* = 0.81) [6,13]. The CAPS is commonly utilized by both clinicians and researchers and has been validated for use in a variety of populations for PTSD assessment [10]. However, the CAPS requires approximately 45–60 min to administer.

### 2.4. Data Analysis

Cronbach’s alphas were computed to assess the internal consistency of the PCL-I/F measures. Pearson’s correlations were conducted to determine the convergent validity of the PCL-I/F, PCL-I, and PCL-F to the CAPS and the CAPS’ subcomponents (e.g., symptom criteria B, C, and D).

## 3. Results

### 3.1. Sample Characteristics

The sample consisted of 92 male veterans with a mean age of 39.15 years (*SD* = 14.24) and a mean of 13.79 years of education (*SD* = 1.93). More than one-half were Caucasian (*n* = 55, 59.80%), and the majority of the remainder were Black (*n* = 22, 23.90%). Of the remaining, four identified as White, Hispanic (4.30%), one identified as Black, Hispanic (1.10%), one identified as American Indian/Alaska Native (1.10%), seven indicated Other (7.60%), and two declined to state (2.20%).

### 3.2. Internal Consistency of the PCL-I/F

The PCL-I/F had strong internal consistency, with Cronbach’s α values of 0.87 for the PCL-I items, 0.86 for the PCL-F items, and 0.93 for the PCL-I/F items, indicating that the items are highly correlated with one another.

### 3.3. Correlations between PCL-I/F and CAPS

Pearson’s correlations yielded significant relationships between the total PCL-I/F score and the total CAPS score, as well as between PCL-I/F and CAPS intensity and frequency subscores, respectively (see Table 1).

**Table 1:** Correlation matrix of PCL-I/F and CAPS.

Measure	1	2	3	4	5	6
1. PCL-I	--					
2. PCL-F	0.72 ***	--				
3. PCL-I/F	0.97 ***	0.89 ***	--			
4. CAPS total score	0.95 ***	0.69 ***	0.70 ***	--		
5. CAPS intensity score	0.73 ***	0.55 ***	0.60 ***	0.90 ***	--	
6. CAPS frequency score	0.68 ***	0.72 ***	0.72 ***	0.95 ***	0.73 ***	--

Note: * *p* < 0.05; ** *p* < 0.01; *** *p* < 0.001; CAPS = Clinician Administered PTSD Scale; PCL-F = PTSD Checklist-Frequency; PCL-I = PTSD Checklist-Intensity; PCL-I/F = PTSD Checklist-Intensity/Frequency.

To assess the convergent validity of the PCL-I, the PCL-I total score and intensity scores for symptom groups B, C, and D were compared to the CAPS total intensity score and its intensity scores for symptom groups B, C, and D, respectively. PCL-I and symptom group B, C, and D scores were significantly correlated with the CAPS intensity score and its respective B, C, and D symptom group scores (see Table 2).

**Table 2:** Correlation matrix of PCL-I and CAPS intensity scores.

Measure	1	2	3	4	5	6	7	8
1. PCL-I score	--							
2. PCL-I symptom group B	0.79 ***	--						
3. PCL-I symptom group C	0.90 ***	0.57 ***	--					
4. PCL-I symptom group D	0.80 ***	0.48 ***	0.60 ***	--				
5. CAPS intensity score	0.62 ***	0.52 ***	0.57 ***	0.45 ***	--			
6. CAPS intensity symptom group B	0.23 *	0.46 ***	0.09	0.10	0.66 ***	--		
7. CAPS intensity symptom group C	0.61 ***	0.34 **	0.71 ***	0.40 ***	0.78 ***	0.19	--	
8. CAPS intensity symptom group D	0.47 ***	0.32 **	0.38 ***	0.49 ***	0.74 ***	0.28 **	0.43 ***	--

Note: * *p* < 0.05; ** *p* < 0.01; *** *p* < 0.001; CAPS = Clinician Administered PTSD Scale; PCL-I = PTSD Checklist-Intensity.

Similar analyses were conducted to examine the convergent validity of the PCL-F. The PCL-F total score and frequency scores for symptom groups B, C, and D were significantly correlated with the CAPS total frequency score and its respective frequency scores for symptom groups B, C, and D (see Table 3).

Finally, analyses were conducted to examine the convergent validity of the PCL-I/F to the CAPS based on symptom criteria B, C, and D. PCL-I/F total score and combined intensity and frequency scores for groups B, C, and D were significantly correlated with the CAPS combined intensity and frequency total scores as well as the CAPS combined scores for groups B, C, and D (see Table 4).

**Table 3:** Correlation matrix of PCL-F and CAPS frequency scores.

Measure	1	2	3	4	5	6	7	8
1. PCL-F	--							
2. PCL-F symptom group B	0.73 ***	--						
3. PCL-F symptom group C	0.90 ***	0.45 ***	--					
4. PCL-F symptom group D	0.86 ***	0.53 ***	0.65 ***	--				
5. CAPS frequency score	0.72 ***	0.52 ***	0.61 ***	0.65 ***	--			
6. CAPS frequency symptom group B	0.33 **	0.58 ***	0.11	0.26 *	0.62 ***	--		
7. CAPS frequency symptom group C	0.62 ***	0.25 *	0.68 ***	0.53 ***	0.86 ***	0.24 *	--	
8. CAPS frequency symptom group D	0.66 ***	0.50 ***	0.49 ***	0.70 ***	0.82 ***	0.40 ***	0.60 ***	--

Note: * *p* < 0.05; ** *p* < 0.01; *** *p* < 0.001; CAPS = Clinician Administered PTSD Scale; PCL-F = PTSD Checklist-Frequency.

**Table 4:** Correlation matrix of PCL-I/F and CAPS frequency scores.

Measure	1	2	3	4	5	6	7	8
1. PCL-I/F	--							
2. PCL-I/F symptom group B	0.78 ***	--						
3. PCL-I/F symptom group C	0.91 ***	0.54 ***	--					
4. PCL-I/F symptom group D	0.86 ***	0.59 ***	0.66 ***	--				
5. CAPS	0.72 ***	0.56 ***	0.64 ***	0.64 ***	--			
6. CAPS intensity and frequency symptom group B	0.30 **	0.57 ***	0.10	0.23 *	0.61 ***	--		
7. CAPS intensity and frequency symptom group C	0.65 ***	0.32 **	0.73 ***	0.51 ***	0.86 ***	0.24 *	--	
8. CAPS intensity and frequency symptom group D	0.58 ***	0.43 ***	0.43 ***	0.67 ***	0.74 ***	0.31 **	0.51 ***	--

Note: * *p* < 0.05; ** *p* < 0.01; *** *p* < 0.001; CAPS = Clinician Administered PTSD Scale; PCL-I/F = PTSD Checklist-Intensity/Frequency.

## 4. Discussion

This pilot study provides initial support that the PCL-I/F is a valid and reliable modification of the PCL. The PCL-I/F, despite modifications in wording and separation of symptom ratings into intensity and frequency determinations, was demonstrated to have both high internal consistency and high correlation with well-validated PTSD psychometric tool, the CAPS. Specifically, both symptom intensity and frequency ratings for the PCL-I/F were demonstrated to have these characteristics. It is thus expected that future research to further develop this instrument and its full psychometric properties will contribute a useful addition to currently available PTSD symptom assessment tools. A brief self-report symptom measure that obtains valid and reliable information on both intensity and frequency of PTSD symptoms providing a more fine-grained analysis of PTSD symptom severity [8] has not been previously available.

A limitation of the findings of this study is that a revision of the *Diagnostic and Statistical Manual of Mental Disorders* criteria into a new edition (5th edition, *DSM-5*) was released after this study was conducted [14]. The diagnostic criteria for PTSD were substantially changed in *DSM-5*. The National Center for PTSD has already released a revised version of the PCL for *DSM-5* [15]. The promising findings from the current study’s revision of the PCL to collect and differentiate intensity and frequency components of posttraumatic symptom severity suggest that additional efforts to further revise this instrument to conform to *DSM-5* criteria will likely also be successful. Additional research to further validate the PCL-I/F, including test-retest reliability testing, updating to *DSM-5* criteria, and extending validation to *DSM-5* criteria, is needed. Moreover, the sample was prohibitively small to permit exploratory factorial analysis (EFA) [16]. Also, the order of administration of the PCL-I and PCL-F was not alternated; therefore, possible order effects are not measurable. Because the PCL-I/F psychometric properties were examined only within a small all-male military veteran population, additional studies will be needed to validate this instrument for other populations to include women and civilians.

## 5. Conclusions

This pilot study developed and examined internal consistency and convergent validity of the PCL-I/F. Despite the noted limitations of this study, the PCL-I/F demonstrated acceptable initial psychometric properties. However, further work is needed to update the instrument to *DSM-5* criteria, test this updated instrument’s properties, fully validate the instrument (including test-retest analysis, divergent validity, and EFA), and test it with different populations. Further revision and validation of this instrument has the potential to provide a more complete understanding of posttraumatic symptoms by assessing both intensity and frequency using a brief self-report symptom measure that has not previously been available.

## Appendix

PCL-I (Intensity) for PCL-I/F

Directions: Circle the number below to the right that indicates **TO WHAT DEGREE** you have been bothered by that problem, related to a stressful military experience, in **the past MONTH**.

Record the stressful military experience: _________________________________________________.

		**Not at all**	**A little bit**	**Moderately**	**Quite a bit**	**Extremely**
1.	Repeated, disturbing *memories*, *thoughts*, or *images* of a stressful military experience?	1	2	3	4	5
2.	Repeated, disturbing *dreams*, of a stressful military experience?	1	2	3	4	5
3.	Suddenly *acting* or *feeling* as if a stressful military experience were *happening again* (as if you were reliving it)?	1	2	3	4	5
4.	Feeling very upset when *something* reminded you of a stressful military experience?	1	2	3	4	5
5.	Having *physical* reactions (e.g., heart pounding, trouble breathing, sweating) when *something reminded you* of a stressful military experience)?	1	2	3	4	5
6.	Avoiding *thinking* about or *talking* about a stressful military experience or avoiding having *feelings* related to it?	1	2	3	4	5
7.	Avoiding *activities* or *situations* because *they reminded you* of a stressful military experience?	1	2	3	4	5
8.	Trouble *remembering important parts* of a stressful military experience?	1	2	3	4	5
9.	Loss of *interest* in activities that you used to enjoy?	1	2	3	4	5
10.	Feeling *distant* or *cut off* from other people?	1	2	3	4	5
11.	Feeling emotionally numb or being unable to have loving feelings for those close to you?	1	2	3	4	5
12.	Feeling as if you *future* will somehow be *cut short*?	1	2	3	4	5
13.	Trouble *falling* or staying *asleep*?	1	2	3	4	5
14.	Feeling irritable or having angry outbursts?	1	2	3	4	5
15.	Having *difficulty* concentrating?	1	2	3	4	5
16.	Being “super alert” or watchful or on guard?	1	2	3	4	5
17.	Feeling *jumpy* or easily startled?	1	2	3	4	5

PCL-F (Frequency) for PCL-I/F

Directions: Below is a list of problems and complaints that people sometimes have in response to stressful experiences. Circle the number below to the right that indicates **HOW OFTEN** you have been bothered by that problem, related to a stressful military experience, in **the past MONTH**.

Record the stressful military experience: _________________________________________________

		**Not at all**	**Once or twice**	**1-2 days a week**	**3-4 days a week**	**Daily or almost every day**
1.	Repeated, disturbing *memories*, *thoughts*, or *images* of a stressful military experience?	1	2	3	4	5
2.	Repeated, disturbing *dreams*, of a stressful military experience?	1	2	3	4	5
3.	Suddenly *acting* or *feeling* as if a stressful military experience were *happening again* (as if you were reliving it)?	1	2	3	4	5
4.	Feeling very upset when *something* reminded you of a stressful military experience?	1	2	3	4	5
5.	Having *physical* reactions (e.g., heart pounding, trouble breathing, sweating) when *something reminded you* of a stressful military experience)?	1	2	3	4	5
6.	Avoiding *thinking* about or *talking* about a stressful military experience or avoiding having *feelings* related to it?	1	2	3	4	5
7.	Avoiding *activities* or *situations* because *they reminded you* of a stressful military experience?	1	2	3	4	5
8.	Trouble *remembering important parts* of a stressful military experience?	1	2	3	4	5
9.	Loss of *interest* in activities that you used to enjoy?	1	2	3	4	5
10.	Feeling *distant* or *cut off* from other people?	1	2	3	4	5
11.	Feeling emotionally numb or being unable to have loving feelings for those close to you?	1	2	3	4	5
12.	Feeling as if you *future* will somehow be *cut short*?	1	2	3	4	5
13.	Trouble *falling* or staying *asleep*?	1	2	3	4	5
14.	Feeling irritable or having angry outbursts?	1	2	3	4	5
15.	Having *difficulty* concentrating?	1	2	3	4	5
16.	Being “super alert” or watchful or on guard?	1	2	3	4	5
17.	Feeling *jumpy* or easily startled?	1	2	3	4	5
